# Re-evaluating suburban green spaces: their role in depression mitigation and social resilience

**DOI:** 10.3389/fpubh.2026.1754959

**Published:** 2026-05-13

**Authors:** Jing Ma, Jiajia Cao, Saisai Wen, Edward A. McBean, Zhengbing Guo, Yuping Wang, Peng Han

**Affiliations:** 1 School of Public Policy and Administration, Xi’an Jiaotong University, Xi’an, China; 2 College of Engineering, University of Guelph, Guelph, ON, Canada; 3 Institute of Social Psychology, School of Humanities and Social Sciences, Xi’an Jiaotong University, Xi’an, China; 4 Department of Otorhinolaryngology-Head and Neck Surgery, The First Affiliated Hospital of Xi’an Jiaotong University, Xi’an, China

**Keywords:** depression mitigation, green infrastructure, mental health, societal resilience, suburban green spaces

## Abstract

**Objective:**

This study investigated how suburban green spaces influence depression and social resilience and compared their effects with those of urban green spaces under varying social and environmental conditions. By evaluating the differential psychological impacts of urban and suburban green spaces in 2013 and 2021, we examined their distinct effects, with robust tests which confirm the reliability of the findings.

**Methods:**

Data from the 2013 and 2021 waves of the Chinese General Social Survey (CGSS) (2,461 respondents) were analyzed by using an Ordinary Least Squares (OLS) regression model.

**Results:**

The results show that suburban green spaces are not significantly associated with reduced depression under normal conditions. However, the psychological benefits related of two types of spaces, namely urban and suburban spaces, become evident when mobility is restricted or social interactions decrease. In contrast, urban green spaces consistently enhance mental health in normal contexts, but their positive effects diminish during periods of limited social contact or elevated environmental stress.

**Conclusion:**

This study clarifies the long-standing debate regarding the effectiveness of suburban green spaces. By utilizing residents’ perceptions rather than objective indicators, it more accurately captures how individuals experience both urban and suburban green areas. The differential findings between 2013 and 2021 reveal the significantly context-dependent nature of green spaces’ impact on mental health. In particular, the research highlights the critical role of suburban green spaces in alleviating depression during periods of heightened social stress or restricted mobility. The inclusion of suburban green spaces in mental health and resilience frameworks is supported by scientific evidence. The findings highlight the importance of suburban green spaces for mental health, particularly under conditions of elevated social stress. These results suggest that improving their accessibility may be beneficial for urban planning, while also potentially supporting broader resilience-related outcomes.

## 1 Introduction

While the rapid advance of global urbanization delivers socioeconomic benefits, urbanization also poses significant challenges to residents’ mental health (1). Factors such as high population density, social pressures, and environmental pollution further exacerbate psychological distress (2–4). By 2019, it was estimated that one in eight individuals worldwide suffered from mental disorders, with anxiety and depression being the most common afflictions (5). These trends have underscored the urgent need for urban strategies that address the psychological challenges of modern living.

As humans possess an inherent affinity for nature (6), research suggests that interactions with green spaces are both effective and affordable, as options to mitigate depression among urban populations (7). Green spaces can be broadly categorized based on their proximity to residents: examples include urban green spaces which are easily accessible, and suburban green spaces which are located farther from city centers (8). Urban green spaces, such as parks, community gardens, and street greenery, are often praised for their accessibility and ability to provide mental relief (9). Studies indicate that exposure to urban green spaces are associated with lower stress levels, reduced anxiety, and improved emotional stability (10). Exposure to urban green spaces has been shown to enhance cognitive function and reduce physiological stress responses, as supported by existing studies (11, 12). However, the extent of these benefits remains debatable. Some researchers argue that urban green spaces may have diminished effects due to overcrowding, pollution, and noise disturbances, which can counteract their intended mental health benefits (13). In contrast, suburban green spaces, such as forests, nature reserves, and large recreational parks, are characterized by a more immersive natural environment, lower human density, and higher biodiversity. These features foster a stronger emotional connection to nature and provide therapeutic effects (14), linking them to lower depression rates, improved cognitive function, and reductions in stress-related biomarkers such as cortisol (15, 16), thereby contributing to enhanced psychological restoration and resilience. However, accessibility and transportation constraints may limit urban residents’ ability to benefit from these environments, raising concerns about their practicality as a widespread mental health intervention (17, 18). Therefore, the relative psychological benefits of different types of green spaces under varying social conditions require further investigation.

Beyond individual wellbeing, green spaces play a crucial role in enhancing societal resilience. Amid increasing urban stressors, environmental challenges, and shifts in social dynamics, access to green spaces provides a psychological buffer, helping individuals regulate emotions, reduce stress hormones, and maintain overall mental stability (19). Additionally, integrating green spaces into urban planning strategies enhances a city’s adaptive capacity, offering residents accessible environments for physical activity, social interaction, and psychological relief (20). Further, research also indicates that well-maintained green spaces improve community cohesion and strengthen social support networks, both of which are critical for fostering long-term urban resilience (21). In view of the above, clarifying the distinct mental health advantages offered by urban and suburban green spaces under varying social conditions is critical for designing resilient and sustainable cities that effectively integrate green infrastructure into long-term urban planning.

Despite ample evidence indicating the beneficial impact of green spaces on residents’ mental health, existing research predominantly focuses on the correlation between mental health and objective green areas, with limited inclusion of individual perceptions. These typically have involved use of remote sensing data to quantify green space and assess its degree of greenness. However, studies have shown that the impact of green space on individuals, whether in suburban or urban areas, ultimately depends on perception. Objective greening and residents’ perceived greenness are not always aligned (22). An area classified as highly green based on objective measures may not necessarily be perceived as such by its residents. Instead, perception may be shaped by specific green vegetation, particularly familiar vegetation that enhances the sense of greenness (23). While objectively present green spaces may benefit health through indirect pathways such as air quality improvement, their primary psychological restorative benefits are typically realized through individual perception and experience. Accordingly, this study focuses on the psychosocial dimension and therefore employs perceived green space as its core metric (24).

Building on this background, rather than relying on remote sensing data to measure greening levels (25), this study utilizes survey responses from 2,461 participants in the 2013 and 2021 Chinese General Social Survey (CGSS) both to assess whether perceived green spaces alleviate depression and to examine the distinct mental health effects of urban and suburban green spaces. This study contributes societal resilience by informing broader discussions of green space planning while differentiating the effects of suburban and urban green environments under varying social conditions. Our findings provide valuable insights for policymakers, urban planners, and public health officials seeking to develop strategic, evidence-based approaches for integrating green infrastructure into sustainable cities to enhance mental wellbeing and societal resilience.

## 2 Materials and methods

### 2.1 Data collection

The data used in this study are from the publicly available Chinese General Social Survey (CGSS) conducted in 2013 and 2021 by the National Survey Research Center of Renmin University of China (This study is based on an analysis of secondary data). Both datasets include residents’ perceptions of green space in their residential areas. The project team successfully navigated numerous challenges posed by the pandemic to complete the survey. This large-scale social sampling survey boasts national representativeness and employs a stratified three-stage probability sampling method. The target population includes urban and rural residents across 31 provinces, autonomous regions, and municipalities (excluding Hong Kong, Macao, and Taiwan). After excluding rural samples (associated with village committees) and samples lacking key information, a total of 2,461 valid samples were retained, with 1,520 from 2013 and 941 from 2021.

### 2.2 Variables

To explore the specific impact of green spaces on depression, this study includes perceived urban green space and perceived suburban green space as independent variables, depression as the dependent variable, and a set of control variables. A diagram illustrating the relationships among variables is provided in the Supplementary Figure 1.

#### 2.2.1 Dependent variable

Mental health is defined in academia through two primary lenses. On one hand, it is viewed positively, characterized as a state of happiness where individuals can realize their abilities, manage normal life stresses, work productively, and contribute to their communities (26). Conversely, life stresses can also be viewed negatively, equating mental health with the absence of psychopathological symptoms (27). This definition assesses mental health based on the presence of mental illness symptoms.

The mental health benefits of green space are grounded in stress reduction theory (28) and attention restoration theory (29, 30). Stress reduction theory posits that contact with nature alleviates work-related stress, while attention restoration theory suggests that such contact can quickly replenish attention resources depleted by work. Both theories highlight the mental health advantages of green space from a negative perspective. Consequently, this study measures the dependent variable of mental health through a negative lens, specifically by assessing respondents’ depression. Respondents were asked, “How often did you feel depressed in the past four weeks?” Responses were coded on a scale from 1 to 5, where 1 indicates “never,” 2 represents “rarely,” 3 means “sometimes,” 4 denotes “often,” and 5 signifies “always.” The average self-rated depression score was 1.94, indicating that most respondents considered their mental health to be at a good level.

#### 2.2.2 Independent variables

Perceived green spaces serve as the independent variable in this study, measured through two questions from the CGSS (Table 1). In this study, the two CGSS items are treated as perceptual indicators of different green space contexts rather than direct objective measures. The first question, “Do you think the forest in your area is seriously damaged?” assesses respondents’ perceptions of suburban green spaces (31, 32). The second question, “Do you think the green spaces in your area is insufficient?” gauges perceptions of urban green spaces (33). Respondents selected the option that best reflected their feelings from a scale of “very serious,” “relatively serious,” “average,” “not too serious”, to “not serious”, corresponding to scores from 1 to 5. A high score indicates a greater perceived sufficiency of green spaces. Accordingly, the analyses focused on perceived green space conditions, which may influence mental health through subjective experience, appraisal, and use. These measures should be interpreted as perception-based proxies rather than exact representations of objective urban or suburban green space quantity or quality. Both perceived suburban and urban green spaces were reported to be at an average level, with perceived suburban green spaces slightly higher than that of perceived urban green spaces.

**Table 1:** Variable definitions and measurements (*N* = 2,461).

Variable	Measurement method	Mean	SD	Min	Max
Depression	How often did you feel depressed in the past 4 weeks on a five-point scale (1: never, 5: always)	1.94	0.94	1	5
Perceived suburban green space	How serious do you think the forest destruction in your area is? 5 points (1: very serious, 5: not serious)	3.15	1.32	1	5
Perceived urban green space	Do you think the green space in your area is insufficient? 5 points (1: very serious, 5: not serious)	3.26	1.32	1	5

#### 2.2.3 Control variables

All models controlled for demographic variables and social determinants of depression. The demographic variables included age, ethnicity, gender, marital status, physical condition, and political status (34). Social determinants of depression encompassed family economic status and education level (35, 36).

Gender is categorized as male (coded as 1) or female (coded as 0). Age is treated as a continuous variable, ranging from 17 to 79 years. Marital status is classified as married (coded as 1) or other (coded as 0). Physical condition is a continuous variable rated from 1 to 5, with higher scores indicating better health. Political status includes members of the Communist Party, Communist Youth League, or Democratic Party (coded as 1) and the masses (coded as 0). Family economic status reflects local income levels, measured as a continuous variable from 1 to 5; higher scores represent economic status above the local average. Ethnicity is classified into ethnic minorities (coded as 1) and Han (coded as 0). Education level is categorized from 1 to 4, where primary school and below is coded as 1, junior high school or equivalent as 2, high school or equivalent as 3, and undergraduate and above as 4.

#### 2.2.4 Statistical analysis

A descriptive analysis was first performed on the dependent, independent, and control variables. Subsequently, we employed the OLS regression model to examine the relationship between perceived green spaces and residents’ depression. This paper conducted OLS regression analyses for two subsamples from 2013 and 2021, respectively, to evaluate whether perceived urban green spaces positively influenced residents’ depression during the pandemic. Models 1–4 detail the analysis results for 2013, while Models 5–8 present the findings for 2021. All analyses were executed using Stata software, version 18. A two-tailed *p* value of less than or equal to 0.1 was considered statistically significant. Prior to conducting statistical analyses, a variance inflation factor (VIF) test was used to assess multicollinearity among variables, with all VIF values being less than 4, indicating no multicollinearity issues in the model described herein.

## 3 Results

### 3.1 Demographic characteristics

Table 2 presents the descriptive statistics for the study sample (*N* = 2,461), demonstrating that this research encompasses a diverse population in terms of gender, age, economic status, and educational background. These data provide a robust foundation for analyzing the relationship between perceived green spaces and depression. Regarding age structure, all respondents fall within the 17–79 age range, with 81.4% aged 17–60 and 18.6% aged 61–79. Additionally, 71% of participants are married. 68.6% of respondents reported their health status as above average. In terms of household economic status, 59.1% perceive their financial situation as average, while 26.4% consider their economic status below average. Concerning educational attainment, 37.5% hold a bachelor’s degree or higher, while the remaining respondents have lower levels of education. In addition, Table 2 presents the specific distributions of gender, marital status, self-rated physical health, political affiliation, and ethnicity. These findings indicate that the study sample is highly representative, allowing the conclusions to accurately reflect the perceptions of different social groups regarding green space and its relationship with depression.

**Table 2:** Demographic characteristics of participants (*N* = 2,461).

Characteristics	Category	*N*	Percentage (%)
Gender	Female	1,197	48.6%
	Male	1,264	51.4%
Age	17–60	2,003	81.4%
	61–79	458	18.6%
Marital status	Married	1,747	71
	Other	714	29
Physical health	Very unhealthy	39	1.6%
	Somewhat unhealthy	178	7.2%
	Average	555	22.6%
	Somewhat healthy	1,015	41.2%
	Very healthy	674	27.4%
Political status	Masses	1,275	51.8
	Communist party (communist youth league) members of democratic parties	1,186	48.2
Family economic level	Far below average	94	3.8
	Below average	649	26.4%
	Average	1,454	59.1%
	Above average	254	10.3%
	Far above average	10	0.4%
Nationality	Minority	154	6.3%
	Han	2,307	93.7%
Education level	Primary school or below	322	13.1%
	Junior high school or equivalent	564	22.9%
	High school or equivalent	652	26.5%
	Bachelor degree or above	923	37.5%
Year	2013	1,520	61.8%
	2021	941	38.2%

### 3.2 Comparison between 2013 and 2021: changes in the impact of perceived suburban green spaces on depression

To explore changes in people’s mental health after undergoing home quarantine and the role of green spaces in this context, this study conducts a comparative analysis of data from 2013 and 2021, revealing the dynamic relationship between perceived green space and depression (see Tables 3 and 4). A key contribution of this analysis lies not only in providing new insights into the mental health benefits of green spaces across different social contexts but also in demonstrating that these effects are context-dependent.

**Table 3:** Results of the effect of perceived green space on self-rated depression in 2013.

Dependent variable: depression	Model 1	Model 2	Model 3	Model 4
Perceived urban green space		−0.028^*^ (0.016)		−0.043^**^ (0.020)
Perceived suburban green space			0.002 (0.016)	0.027 (0.020)
Gender	−0.011 (0.045)	−0.013 (0.045)	−0.011 (0.045)	−0.015 (0.045)
Age	−0.006^***^ (0.002)	−0.005^***^ (0.002)	−0.006^***^ (0.002)	−0.005^***^ (0.002)
Marital status	0.001 (0.055)	−0.001 (0.055)	−0.001 (0.055)	−0.003 (0.055)
Physical condition	−0.386^***^ (0.026)	−0.383^***^ (0.026)	−0.386^***^ (0.026)	−0.383^***^ (0.026)
Political status	−0.013 (0.002)	−0.007 (0.054)	−0.013 (0.054)	−0.007 (0.035)
Family economic status	−0.002 (0.035)	−0.001 (0.035)	−0.002 (0.035)	−0.001 (0.035)
Nationality	0.235^***^ (0.084)	0.228^***^ (0.085)	0.235^***^ (0.084)	0.223^***^ (0.085)
Education level	−0.071^***^ (0.026)	−0.074^***^ (0.026)	−0.071^**^ (0.026)	−0.073^***^ (0.026)
Constant	3.917^***^ (0.183)	3.981^***^ (0.187)	3.911^***^ (0.189)	3.942^***^ (0.189)
*N*	1,520	1,520	1,520	1,520
*R* ^2^	0.156	0.158	0.156	0.159

^*^p < 0.1, ^**^p < 0.05, ^***^p < 0.01, standard errors in parentheses.

**Table 4:** Results of the effect of perceived green space on self-rated depression in 2021.

Dependent variable: depression	Model 5	Model 6	Model 7	Model 8
Perceived urban green space		−0.040 (0.025)		−0.001 (0.031)
Perceived suburban green space			−0.070^***^ (0.026)	−0.069^**^ (0.033)
Gender	−0.127^**^ (0.060)	−0.126^**^ (0.060)	−0.123^**^ (0.059)	−0.123^**^ (0.059)
Age	−0.005^**^ (0.002)	−0.005^**^ (0.002)	−0.005^**^ (0.002)	−0.005^**^ (0.002)
Marital status	−0.073^*^ (0.067)	−0.073 (0.067)	−0.073 (0.066)	−0.073 (0.067)
Physical condition	−0.386 ^***^ (0.033)	−0.382^***^ (0.033)	−0.383^***^ (0.033)	−0.383^***^ (0.033)
Political status	0.134 (0.088)	0.134 (0.088)	0.127 (0.088)	0.127 (0.088)
Family economic status	−0.093^**^ (0.041)	−0.089^**^ (0.041)	−0.084^**^ (0.041)	−0.084^**^ (0.041)
Nationality	−0.246^*^ (0.142)	−0.246^*^ (0.142)	−0.218 (0.142)	−0.218 (0.142)
Education level	0.001 (0.033)	−0.004 (0.034)	−0.007 (0.034)	−0.007 (0.034)
Constant	3.857^***^ (0.261)	3.982^***^ (0.272)	4.091^***^ (0.275)	4.092^***^ (0.277)
*N*	941	941	941	941
*R* ^2^	0.159	0.161	0.165	0.165

^*^p < 0.1, ^**^p < 0.05, ^***^p < 0.01, standard errors in parentheses.

#### 3.2.1 2013: the dominant role of urban green spaces in mental health

In 2013, perceived urban green spaces significantly alleviated depression (*B* = −0.028, *p* < 0.1, Model 2; *B* = −0.043, *p* < 0.05, Model 4). This means that individuals who perceived more urban green spaces reported lower depression levels, aligning with previous research indicating that urban green spaces improve mental health due to their high accessibility and ease of daily use.

Model 2, which includes only Perceived urban green space as the main explanatory variable, demonstrates a significant mitigating effect on residents’ depression. In contrast, Model 3, which includes only Perceived suburban green space, yields statistically non-significant results. Model 4 (including both perceived urban and suburban green spaces) shows the highest explanatory power (*R*^2^ = 0.159), yet perceived suburban green spaces remain insignificant, reinforcing the dominant role of urban green spaces in alleviating depression in 2013. This finding is consistent with previous studies showing that exposure to natural environments can restore cognitive resources and reduce stress (37, 38). Additionally, urban green spaces are often better maintained and equipped with infrastructure (e.g., walking paths, seating areas, lighting), enhancing user experience and making these spaces more attractive (39). These features establish urban green spaces as an essential mental health resource, which was particularly evident in the 2013 dataset. Furthermore, this conclusion may also be linked to the higher accessibility of urban green spaces compared to suburban ones.

Moreover, the 2013 survey data indicate that perceived suburban green spaces had no significant effect on depression, further supporting the priority role of urban green spaces.

#### 3.2.2 2021: the rising role of suburban green spaces in depression mitigation

By 2021, perceived suburban green spaces emerged as a significant factor in reducing depression (*B* = −0.070, *p* < 0.01, Model 7; *B* = −0.069, *p* < 0.05, Model 8), whereas perceived urban green spaces showed no significant effect.

This shift indicates that the mental health benefits of suburban green spaces become more prominent as social environments and residents’ needs evolved. These findings challenge conventional perspectives which generally assume that urban green spaces are always superior in promoting mental health due to their higher accessibility (39). Instead, this study suggests that in specific social contexts, suburban green spaces may play a more critical role in mental health.

### 3.3 Robustness test

To verify the dominant roles of urban and suburban green spaces in mental health for 2013 and 2021, this paper conducted subgroup regressions by dividing the selected control variables into two groups. For 2013, the first four variables (Gender, Age, Marital Status, Physical Condition) are selected, while for 2021, the latter four variables (Political Status, Family Economic Status, Nationality, Education Level) are used for robustness testing. Additionally, logistic regression was applied for validation in both years. The results are presented in Tables 5 and 6.

**Table 5:** Robustness test of urban green space’s dominant role in mental health.

Dependent variable: depression	Model 9 Gender = 1	Model 10 Age < 61	Model 11 Marital status = 1	Model 12 Physical condition = 3, 4, 5	Model 13 Logistic regression
Perceived urban green space	−0.050^*^ (0.030)	−0.040^*^ (0.021)	−0.058^**^ (0.022)	−0.051^**^ (0.020)	−0.144^**^ (0.059)
Perceived suburban green space	0.035 (0.030)	0.033 (0.021)	0.036 (0.022)	0.031 (0.020)	0.021 (0.058)
Controls	√	√	√	√	√
*N*	682	1,287	1,136	1,398	1,520
*R* ^2^	0.143	0.146	0.165	0.096	0.108

^*^p < 0.1, ^**^p < 0.05, ^***^p < 0.01, standard errors in parentheses. Models 9–12 are based on stratified sampling methods, with Model 9 representing the female population, Model 10 representing the non-retired population, Model 11 representing the married population, and Model 12 representing the population in good physical condition. Model 13 employs a different estimation method, namely logistic regression.

**Table 6:** Robustness test of suburban green space’s dominant role in mental health.

Dependent variable: depression	Model 14 Political status = 0	Model 15 Family economic status = 1, 2, 3	Model 16 Nationality = 0	Model 17 Education level = 1, 2, 3	Model 18 Logistic regression
Perceived urban green space	0.006 (0.035)	−0.003 (0.034)	−0.009 (0.033)	0.036 (0.039)	−0.024 (0.082)
Perceived suburban green space	−0.061^*^ (0.036)	−0.058^*^ (0.035)	−0.069^**^ (0.034)	−0.073^*^ (0.041)	−0.164^*^ (0.086)
Controls	√	√	√	√	√
*N*	801	836	899	599	941
*R* ^2^	0.166	0.171	0.166	0.214	0.092

^*^p < 0.1, ^**^p < 0.05, ^***^p < 0.01, standard errors in parentheses. Models 14–17 are based on stratified sampling methods, with Model 14 representing the population with a political status of Masses, Model 15 representing the population with a family economic status of medium to below average, Model 16 representing the population with a nationality of non-minority, and Model 17 representing the population with an education level of primary or secondary. Model 18 employs a different estimation method, namely logistic regression.

As shown in Table 5, after controlling for other variables, all four subsamples (Gender = Female, Age < 61, Marital Status = Married, Physical Condition = Average, Somewhat healthy, Very healthy) demonstrated that Perceived urban green space significantly reduced depression, whereas perceived suburban green space showed no significant effect. This aligns with the original regression, confirming the reliability of the conclusion.

Similarly, the regression results for the four subsamples in Table 6 (Political Status = Masses, Family Economic Status = Medium to Below Average, Nationality = Non-minority, Education Level = Primary or Secondary) also match the original regression. This indicates that from 2013 to 2021, the depression-reducing effect of perceived urban green space shifted from significant to insignificant, while perceived suburban green space transitioned from insignificant to significant. These findings robustly support the validity of Model 8 in Table 4.

### 3.4 Heterogeneity analysis

To investigate the impact of perceived green spaces on mental health across different population groups, this study conducted heterogeneity analyses based on age (young, middle-aged, older adults), economic status (low, medium, high), and education level (higher education, non-higher education).

The 2013 heterogeneity analysis (Table 7) revealed that the depression-alleviating effect of urban green spaces was most pronounced among middle-aged groups (*B* = −0.090, *p* < 0.01), those with lower household economic status (*B* = −0.082, *p* < 0.05), and non-higher education subgroups (*B* = −0.059, p < 0.05). In contrast, suburban green spaces showed no significant effects in most demographic groups. This finding aligns with the main regression results, reinforcing the supportive role of accessible urban green spaces, under normal social conditions, for vulnerable populations such as the middle-aged (who bear substantial social and familial pressures), economically disadvantaged groups, and those with limited education.

**Table 7:** Heterogeneity analysis of urban green space’s dominant role in mental health.

Dependent variable: depression	Model 19: Age			Model 20: Family economic status			Model 21: Education level	
	<35	35–59	>59	<3	=3	>3	<4	=4
Perceived urban green space	0.025 (0.034)	−0.090^***^ (0.029)	−0.044 (0.052)	−0.082^**^ (0.039)	−0.008 (0.025)	−0.099 (0.061)	−0.059^**^ (0.027)	−0.019 (0.029)
Perceived suburban green space	0.030 (0.033)	0.043 (0.029)	−0.018 (0.051)	0.072^*^ (0.040)	−0.001 (0.025)	0.038 (0.063)	0.019 (0.027)	0.039 (0.029)
Controls	√	√	√	√	√	√	√	√
*N*	487	729	268	426	935	159	939	581
*R* ^2^	0.153	0.165	0.224	0.248	0.133	0.125	0.169	0.122

^*^p < 0.1, ^**^p < 0.05, ^***^p < 0.01, standard errors in parentheses.

By 2021 (Table 8), the pattern underwent a number of changes. The mental health benefits of suburban green spaces became evident, showing marginally significant depression-reducing effects among the older adults (*B* = −0.113, *p* < 0.1), groups with medium economic status (*B* = −0.101, *p* < 0.05), and those with lower education levels (*B* = −0.073, *p* < 0.1). Meanwhile, the positive effects of urban green spaces became non-significant for most groups in 2021. Notably, among the older adults (>59 years), urban green space perception showed an unexpected positive association with depression levels (*B* = 0.101, *p* < 0.05), possibly reflecting this group’s heightened concerns about using communal urban spaces or accessibility barriers during the pandemic.

**Table 8:** Heterogeneity analysis of suburban green space’s dominant role in mental health.

Dependent variable: depression	Model 22: Age			Model 23: Family economic status			Model 24: Education level	
	<35	35–59	>59	<3	=3	>3	<4	=4
Perceived urban green space	−0.032 (0.064)	−0.038 (0.049)	0.101^*^ (0.057)	−0.015 (0.053)	0.010 (0.043)	0.041 (0.090)	0.036 (0.038)	−0.070 (0.053)
Perceived suburban green space	−0.010 (0.067)	−0.061 (0.051)	−0.113^*^ (0.061)	−0.008 (0.056)	−0.101^**^ (0.044)	−0.161 (0.114)	−0.073^*^ (0.041)	−0.039 (0.054)
Controls	√	√	√	√	√	√	√	√
*N*	293	393	234	317	519	105	599	342
*R* ^2^	0.091	0.179	0.314	0.261	0.105	0.212	0.214	0.132

^*^p < 0.1, ^**^p < 0.05, ^***^p < 0.01, standard errors in parentheses.

The heterogeneity analysis underscores that the mental health benefits of green spaces are not uniform but are jointly moderated by demographic characteristics and broader social contexts.

## 4 Discussion

### 4.1 Main findings

This study employed the Ordinary Least Squares (OLS) regression model with survey data from 2013 and 2021 covering 2,461 respondents, providing robust evidence that the perceived benefits of suburban green spaces in alleviating depressive symptoms are progressively increasing. Notably, the analysis reveals that these perceived benefits are amplified by specific contextual factors, such as the growing need for quiet and private spaces, evolving lifestyles, and the pervasive influence of social media. It also identifies significant associations between perceived green spaces and both psychological connectedness to nature and residential wellbeing, offering empirical support for prioritizing suburban green spaces in future urban planning. Therefore, this study offers insightful implications for future research examining the psychological, social, and urban development dimensions of green space.

### 4.2 The role of perceived suburban green spaces in specific social contexts

Beyond the objective factors mentioned earlier, in specific social contexts residents’ perception of suburban green spaces may be significantly enhanced, thereby potentially contributing to the alleviation of depressive symptoms. In this study, the 2021 CGSS wave was collected during the COVID-19 pandemic, when China implemented a range of public health measures (e.g., stay at home guidance, mobility and travel restrictions, and heightened concern about infection risks in crowded settings). Such conditions may have shaped residents’ daily routines, access to public spaces, and the ways they perceived and used different types of green spaces, providing useful context for interpreting the differences observed between 2013 and 2021.

Firstly, in certain circumstances, the demand for quiet and private spaces tends to increase. When high-density urban environments fail to provide adequate leisure space, urban green spaces may experience overcrowding, leading to a decline in environmental quality and a diminished user experience (40). Consequently, residents might develop negative perceptions of these spaces, such as increased congestion and reduced comfort, which could reduce usage frequency and diminishes their mental health benefits (41). In contrast, suburban green spaces, with their larger areas, more diverse natural landscapes, and greater biodiversity, are often perceived as safer and more comfortable psychological refuges, making them potentially crucial stress-relief resources in certain situations (42). For example, both during and after the COVID-19 pandemic, concerns about infection risk in crowded public settings may have amplified residents’ negative perceptions of urban green spaces when such spaces were associated with density, regulation, or restricted movement. In contrast, suburban green spaces may have been perceived as lower-risk and more restorative environments, thereby offering greater psychological relief under conditions of heightened stress and constrained mobility.

Secondly, changes in lifestyle patterns can also influence the perception of green spaces. In the post-COVID-19 era, with the rise of remote work and increased time spent at home, urban green spaces may become of greater interest related to association of work-related stress and daily responsibilities, possibly weakening their function as relaxation spaces. By contrast, suburban green spaces, being physically distant from urban noise and work pressures, are more likely to be perceived as pure recreational areas, further enhancing their role in psychological regulation.

Additionally, media representation and social media discourse can shape public perceptions of green spaces. In specific social contexts, social media platforms may highlight the advantages of suburban green spaces, emphasizing their benefits in reducing crowd exposure and providing a restorative natural environment (43). This reinforcement could enhance residents’ recognition of their mental health, increasing their preference for suburban green space. Media focus on the therapeutic power of nature and outdoor safety collectively constructs a social narrative during public health crises like the COVID-19 pandemic, which further anchors suburban green spaces as an effective psychological resource for coping with collective stress.

### 4.3 Perceived suburban green spaces, environmental satisfaction, and their impact on depression

The mental health dimensions of perceived green spaces are likely influenced not only by their physical attributes (e.g., size, vegetation type) but also by individual perceptions, social environments, and patterns of use. Research indicates that even within the same community, individuals may perceive green spaces differently, leading to varying effects on their mental health (44). This subjective perception could shape how individuals experience green spaces, thereby moderating their depressive symptoms.

Studies often show that perceived high-quality green spaces are strongly associated with greater environmental satisfaction and residential wellbeing. When individuals positively evaluate the green spaces in their residential environment, they are more likely to experience psychological relaxation, which in turn reduces the accumulation of negative emotions in daily life. Additionally, high-quality green environments contribute to better cognitive restoration, such as reducing fatigue and enhancing emotional stability, which is broadly consistent with existing research on the restorative effects of natural environments.

Furthermore, research suggests that perceived green spaces can foster stronger emotional connections with nature, which may serve as a critical psychological resource for stress regulation and depression mitigation. Individuals with higher levels of nature connectedness generally exhibit more positive emotions and lower levels of anxiety and depression (45). This finding supports the notion that green spaces function not only as physical environments but also as psychological assets that support emotional wellbeing.

### 4.4 The role of suburban green spaces in societal resilience and urban planning integration

This study concludes that green spaces, especially suburban green spaces, can mitigate individual depression levels under specific conditions. As this conclusion is drawn from a demographically diverse sample (from CGSS), the findings suggest a potential generalizability of the green space effect. Accordingly, when prevalent psychological risks within a society are moderated by such environmental resources, social stability can be strengthened, thereby contributing to enhanced societal resilience. Consequently, green spaces are increasingly recognized to serve not only as buffers for mental health but also as essential components of societal resilience. With modern societies facing growing environmental challenges, increasing urban stress, and evolving socio-economic pressures, urban planning must move beyond conventional infrastructure development to incorporate mental health support and social adaptability into planning frameworks (46).

Research suggests that urban parks, greenways, and nature reserves can play a crucial role in psychological resilience and post-crisis recovery (47). In addition to their therapeutic benefits, green spaces contribute significantly to community cohesion and social stability. For example, green spaces can be repurposed as temporary shelters in post-disaster recovery, providing safe environments for displaced populations while facilitating mutual community assistance and psychological support. These findings indicate that green spaces should be systematically integrated into urban emergency management and long-term planning [in agreement with (48)].

To maximize the benefits of green spaces, future urban planning should focus on several key areas. First, enhancing accessibility is widely considered essential to ensure that both urban and suburban residents can conveniently access green spaces and fully experience their mental health benefits. Second, optimizing suburban green spaces development is often viewed as crucial for improving environmental quality, infrastructure, and safety measures. Third, integrating suburban green spaces into emergency management systems can ensure they serve as critical psychological support resources during times of crisis. Finally, increasing public awareness through education and outreach programs can encourage more residents to recognize and utilize the mental health benefits of green spaces. By implementing these strategies, urban and suburban green spaces can be expected to contribute not only to individual mental health but also to broader societal resilience and stability.

### 4.5 The moderating role of demographic characteristics: insights from heterogeneity analysis

The heterogeneity analysis in Section 3.4 reveals demographic variations in the benefits of green spaces. Findings from 2013 indicate that urban green spaces were particularly beneficial for middle-aged, economically disadvantaged, and low-income groups. This can be attributed to the importance of easily accessible and well-integrated green infrastructure for populations experiencing daily stressors such as work pressure and financial constraints.

By 2021, the mental health effects of suburban green spaces had become more pronounced among demographic groups similar to those observed in 2013. This shift is particularly revealing in its implications. This suggests that during periods of increased social stress and restricted mobility, the quality and immersive nature of green environments may outweigh mere proximity. For groups facing limited living space and higher baseline stress, access to expansive, natural suburban areas could provide a unique psychological buffer that urban parks, often perceived as crowded or regulated, may not offer. Additionally, the unexpected positive correlation between urban green space and depression levels among the older adults in 2021 warrants attention, potentially reflecting this group’s concerns about infection risks in public urban spaces following the COVID-19 pandemic (49).

## 5 Conclusion

This study provides new empirical evidence on the relationship between perceived green spaces and depression, emphasizing the critical role of subjective perception in shaping mental health outcomes. By leveraging nationally representative data from the 2013 and 2021 waves of the Chinese General Social Survey (CGSS), this research offers a longitudinal perspective on the evolving psychological impacts of urban and suburban green spaces.

A key contribution of this study is the differentiation between the mental health effects of urban and suburban green spaces across varying social contexts. Under normal social conditions, urban green spaces demonstrate a strong protective effect against depression, likely due to their high accessibility and integration into daily routines. However, in periods of heightened societal stress, suburban green spaces emerge as critical psychological refuges, offering higher environmental quality, greater perceived safety, and deeper restorative experiences. Meanwhile, the mental health benefits of green spaces are not uniform but are significantly shaped by individual characteristics, including age and socioeconomic status. This context-dependent role of green spaces challenges the traditional assumption that urban green spaces is universally more beneficial, highlighting the importance of flexibility in green space planning.

By incorporating nationwide empirical data, this study provides robust scientific support for integrating suburban green spaces into mental health and resilience frameworks. It advances a context-sensitive approach to urban and environmental planning, advocating for a more adaptive, inclusive, and resilient model of green infrastructure development. These findings hold significant implications for policymakers and urban planners, reinforcing the need to optimize both urban and suburban green spaces to maximize their mental health benefits across different social and environmental conditions.

This study has several limitations that should be considered when interpreting the findings. Although the comparison between 2013 and 2021 provides valuable insights into shifts in social stress contexts, the lack of a unified quantitative definition and classification framework for high social stress periods may limit the generalizability and precise differentiation of conclusions across stress scenarios of varying types and intensities. And due to constraints in data availability, the study primarily relied on respondents’ subjective perceptions, without incorporating objective indicators. Consequently, it remains difficult to disentangle the independent contributions of subjective and objective factors to mental health or elucidate their potential interactions. Future research could benefit from expanding the temporal scope to include multiple pandemic phases and a wide range of social contexts, enabling a more accurate understanding of evolving trends in residents’ green space needs and mental health status.

## Data Availability

The datasets presented in this article are not readily available because the data used in this study were obtained from a publicly available source, the official website of the Chinese General Social Survey (CGSS, http://cgss.ruc.edu.cn/). These datasets are openly accessible and can be downloaded directly according to individual research needs. Requests to access the datasets should be directed to CGSS, http://cgss.ruc.edu.cn/.
