# Supplementary material for: Re-evaluating suburban green spaces: their role in depression mitigation and social resilience
Source: Front Public Health. 2026 May 13;14:1754959. doi: 10.3389/fpubh.2026.1754959 (PMC13212202; doi:10.3389/fpubh.2026.1754959)
Supplement: Supplementary file 1 [file Image_1.pdf]

Supplementary Material

1 Supplementary Figures

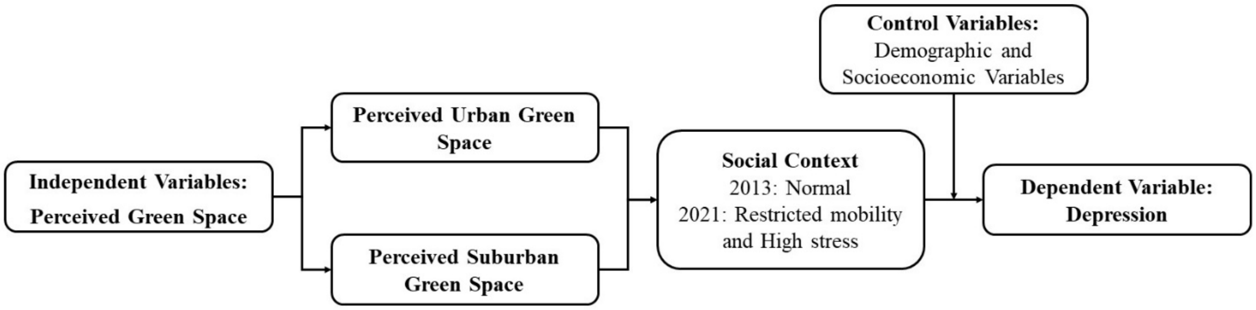

Supplementary Figure 1. Relationships among key variables in the study
